# A large verrucous plaque on the buttocks: a case report of an atypical presentation of tuberculosis verrucosa cutis

**DOI:** 10.11604/pamj.2020.37.131.26150

**Published:** 2020-10-06

**Authors:** Yulia Asmarani, Rizki Citra Mulia, Anni Adriani

**Affiliations:** 1 Department of Dermatology and Venereology, Faculty of Medicine, Universitas Hasanuddin, South Sulawesi, Makassar, Indonesia,; 2 Dr. Wahidin Sudirohusodo Hospital, South Sulawesi, Makassar, Indonesia

**Keywords:** Atypical, annular hyperkeratotic plaque, central healing, tuberculosis verrucosa cutis

## Abstract

Although the incidence of cutaneous tuberculosis (TB) has declined among all cases in dermatology outpatient settings, atypical cases are still being reported worldwide. These atypical forms can imitate other conditions hence delaying the diagnosis and increased morbidity. We report a case of an atypical presentation of extensive Tuberculosis Verrucosa Cutis (TVC) on the buttocks presenting as an annular hyperkeratotic plaque with central healing. Suspicious clinical manifestations and histopathological features followed by an excellent response with antituberculosis therapy confirmed the diagnosis of TVC. Recalcitrant chronic lesions even with adequate standard treatment should raise suspicion of cutaneous TB, especially in endemic areas.

## Introduction

Tuberculosis (TB) is caused by *Mycobacterium tuberculosis* and still considered as a leading cause of infections especially in developing countries. These countries have the greatest burden of the disease, whereas China, India and Indonesia alone accounted for 45% of global cases in the newly issued global TB report [1]. Cutaneous TB is uncommon, only 1% to 2% of all extrapulmonary TB cases showed cutaneous involvement [1]. Cutaneous TB is more prevalent in tropical regions with hot and humid climate, such as Indonesia, in which it parallels with pulmonary TB incidence. In 2018, Indonesia was the third country with the largest number of TB cases worldwide [2,3]. True cutaneous TB can be further divided into: exogenous TB caused by direct inoculation (TB chancre and TB verrucosa cutis), endogenous TB due to direct spread or auto-inoculation (scrofuloderma, orificial TB and some lupus vulgaris) and hematogenous transmission (lupus vulgaris, tuberculous gumma and acute military TB) [1]. Out of all cases of cutaneous TB, TVC is the most common form of cutaneous TB in Asia. It occurs as a result of direct inoculation on the skin of a patient who was previously infected and has a moderate to a high degree of immunity [4]. Inoculation can occur at sites of minor wounds or, rarely, from the patient´s sputum. In a low socioeconomic environment, children are mostly infected by playing on the ground which may be contaminated with TB sputum [3,4].

Although the incidence of cutaneous TB has declined among all cases in dermatology outpatient settings, atypical forms are still being reported worldwide. Rarely, the lesion may become massive with papillomatous infiltration and strong consistency, with relatively soft areas. Sometimes the clinical resemblance to warts, hypertrophic lichen planus, oriental pain, chromoblastomycosis, etc. can be difficult to diagnose. The psoriasiform, sporotrichoid and keloidal appearance of TVC has also been described and can sometimes even clinically mimic lupus vulgaris [5]. Thus, high suspicion index is needed when encountering suspicious lesions. This report presents a case of an atypical TVC presenting as large verrucous plaque that had been neglected for two years.

## Patient and observation

**Figure 1:**
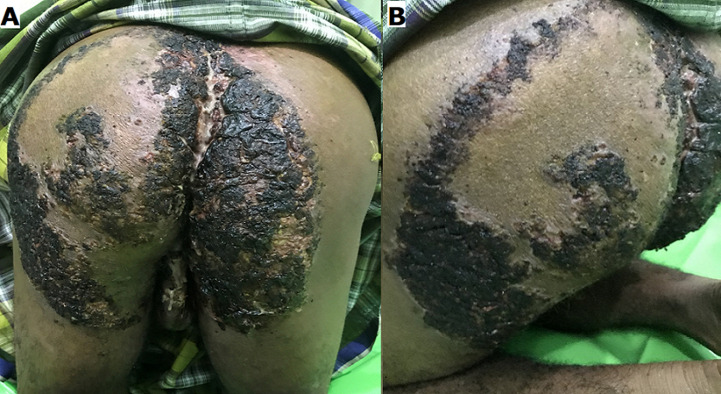
A, B) extensive verrucous plaque covered in dark dense crust over the gluteus, thigh and scrotum with an irregular border; fissures were seen over the dense crust and multiple ulcers

A 35-year-old male was referred to our outpatient clinic with a large verrucous plaque and malodorous purulent discharge on the buttocks, thigh and scrotum. The lesion started two years ago as small, mildly pruritic and multiple plaques that were limited to the buttocks. Some painful and itchy nodules then developed over the lesions in the last five months. The nodules ulcerated and extended with foul-smelling seropurulent discharge. The lesions thickened and expanded rapidly covering the left buttock, thigh and scrotum, forming an annular hyperkeratotic plaque with central healing. The patient had been treated in a local hospital with antifungal and antibiotic therapy without significant improvement. There was no history of previous trauma. The patient worked in an oil palm plantation and often wore shorts and sat on the ground when working. The patient had a history of untreated cough and night sweats without fever in the past two years. There was no family nor household history of TB. Dermatological examination showed extensive verrucous plaque covered in dark dense crust over the buttocks, thigh and scrotum with an irregular border. Fissures and multiple ulcers were seen over the dense crust with foul-smelling seropurulent discharge (Figure 1). Routine blood workups were performed, with liver function, renal function and blood sugar levels were within normal limits. His leucocyte was slightly raised to 14.580/μl. Enzyme-linked immunosorbent assay (ELISA) for HIV was nonreactive. Mantoux test was positive and chest radiograph revealed extensive pulmonary TB.

**Figure 2:**
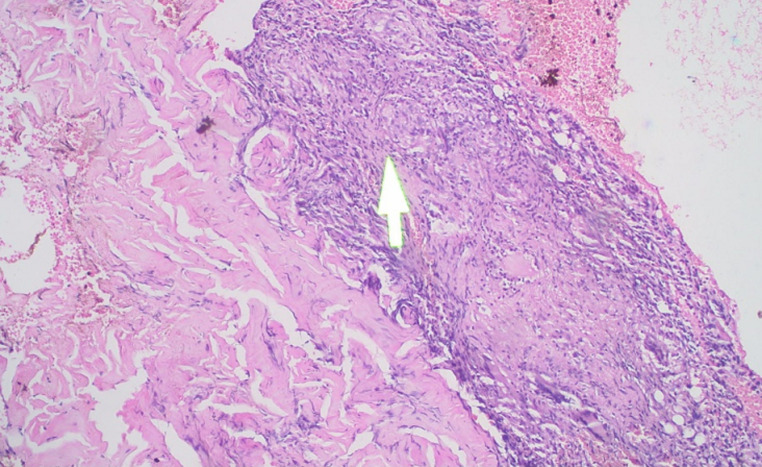
epithelioid granulomatous inflammation surrounding the caseous necrosis (arrow) along with several Langerhans cells in the dermis and many lymphocytic infiltrates

**Figure 3:**
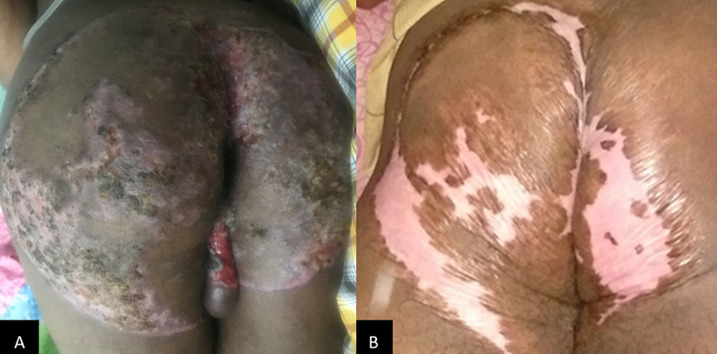
A) at two months follow-up, the skin lesions showed significant improvement; B) the lesions were completely resolved after four months of therapy leaving atrophic scars

Acid Fast Bacilli (AFB) were not found in the sputum specimen, but The GeneXpert was able to detect *M. tuberculosis* that was sensitive to rifampicin. Pus culture was performed and revealed *Escherichia coli* colonization. Skin biopsy of the lesion was performed and histopathology analysis showed marked pseudoepitheliomatous hyperplasia of epidermis, epithelioid granulomatous inflammation surrounding the caseous necrosis along with several Langerhans cells in the dermis and many lymphocytic infiltrates (Figure 2). AFB staining using Ziehl-Neelsen and culture on Lowenstein-Jensen medium were negative. Fungal infection was ruled out by negative potassium hydroxide (KOH) examination from the lesion skin scraping and negative Periodic Acid-Schiff (PAS) stain from the skin biopsy specimen. A multidisciplinary approach by referring to the Pulmonology department was carried out and anti-tuberculosis treatment (ATT) regimen 1 consisted of two-month intensive phase (Rifampicin 450mg, Isoniazid 225mg, Pyrazinamide 1200mg, Ethambutol 825mg daily) followed by four-month continuation phase (Rifampicin 450mg, Isoniazid 450mg three times a week) was given. At two-month follow-up, the skin lesions showed significant improvement and the lesions were completely resolved after four months of therapy leaving atrophic scars (Figure 3).

## Discussion

Clinically, TVC is commonly reported at sites prone to trauma such as the distal extremities, especially on the hands and to a lesser extent, the buttocks [5,6]. The occurrence of TVC following injury has been well documented in several reports [5,7]. The inflicting trauma may only be trivial or accidental [7] which makes the patient unaware of it; this was likely the case in our patient, was thought to be related to the patient's habit of wearing shorts and sitting on the ground while working that facilitated the inoculation of *M. tuberculosis* through contaminated soil. Following the confirmation of cutaneous TB, it is essential to search for extracutaneous foci of TB by urine, blood and sputum samples, thorax X-ray or computed tomography (CT) scan of the chest; and bone scans [8]. Although based on the chest X-ray examination in our case, an active pulmonary TB was confirmed, cases of TVC caused by autoinoculation from sputum itself are very rare. So far only one case of TVC caused by sputum autoinoculation has ever been reported [4]. In addition, AFB was not found in the sputum of our patient, although GeneXpert was positive for *M. tuberculosis*.

The diagnosis of cutaneous TB often poses a challenge to clinicians. The atypical lesion morphology in this case complicated the diagnosis and required high suspicion index. The clinical presentation of this case resembled cutaneous deep mycosis such as chromoblastomycosis. However, no fungal elements were found in skin scrapings, culture and histopathology and there was history of failure with previous antifungal treatment. Taken together, these findings ruled out the involvement of fungal infection in this case. Long-term untreated lesions accompanied by bacterial superinfection led to an atypical presentation in our case. To our knowledge, only study has ever reported TVC presenting as annular hyperkeratotic plaque as observed in our case [5]. There are several cutaneous diseases that have a histopathological presentation of granulomas such as cutaneous leishmaniasis, tuberculoid leprosy, superficial granulomatous pyoderma, cutaneous sarcoidosis, lupus miliaris disseminatus faciei and chromomycosis. However, the characteristic feature of TB infections is tubercle, an accumulation of epithelioid histocytes with surrounding Langhans-type giant cells and a varying amount of central caseating necrosis, surrounded by a rim of lymphocytes and monocytes, which were present in this case [3]. Although these descriptions are commonly found in cutaneous TB, they are only auxiliary in diagnosing cutaneous TB.

TVC is a type of cutaneous TB with histopathological characteristics of granulomas with caseous necrosis [9]. The diagnosis relies on the demonstration of *M. tuberculosis* in smears or biopsies and by the culture of the organism. However, because most types of cutaneous TB are paucibacillary, it is often difficult to demonstrate or grow the organism. The use of the polymerase chain reaction (PCR) to detect mycobacterial DNA raises the possibility of higher diagnostic yield with higher sensitivity. Unfortunately, the availability of PCR especially in developing countries is still problematic; hence in practice dermatologists often have to rely on a therapeutic trial of ATT to confirm the diagnosis in difficult cases [10]. A review article suggests that when PCR is not available, therapeutic test using ATT may be considered [6]. In this case, although *M. tuberculosis* was not found, clinical suspicion, typical histopathological examination and response to ATT confirmed the diagnosis of TVC.

## Conclusion

This case showed that cutaneous TB may present with various clinical presentations. Thus, chronic lesions recalcitrant to adequate standard treatment should raise suspicion of cutaneous TB, especially in endemic areas.
